# Differentiation of low and high grade renal cell carcinoma on routine MRI with an externally validated automatic machine learning algorithm

**DOI:** 10.1038/s41598-020-76132-z

**Published:** 2020-11-11

**Authors:** Subhanik Purkayastha, Yijun Zhao, Jing Wu, Rong Hu, Aidan McGirr, Sukhdeep Singh, Ken Chang, Raymond Y. Huang, Paul J. Zhang, Alvin Silva, Michael C. Soulen, S. William Stavropoulos, Zishu Zhang, Harrison X. Bai

**Affiliations:** 1 Department of Diagnostic Imaging, Rhode Island Hospital, Alpert Medical School of Brown University, Providence, RI 02905 USA; 2 Department of Radiology, The Second Xiangya Hospital, Central South University, Changsha, China; 3 Division of Interventional Radiology, Department of Radiology, Hospital of the University of Pennsylvania, Philadelphia, PA USA; 4 Department of Radiology, Mayo Clinic, Phoenix, AZ USA; 5 Athinoula A. Martinos Center for Biomedical Imaging, Department of Radiology, Massachusetts General Hospital, Boston, MA USA; 6 Department of Radiology, Brigham and Women’s Hospital, Boston, MA USA; 7 Department of Pathology, Hospital of the University of Pennsylvania, Philadelphia, PA USA; 8 School of Computer Science and Engineering, Central South University, Changsha, Hunan China

**Keywords:** Diagnosis, Machine learning

## Abstract

Pre-treatment determination of renal cell carcinoma aggressiveness may help guide clinical decision-making. We aimed to differentiate low-grade (Fuhrman I–II) from high-grade (Fuhrman III–IV) renal cell carcinoma using radiomics features extracted from routine MRI. 482 pathologically confirmed renal cell carcinoma lesions from 2008 to 2019 in a multicenter cohort were retrospectively identified. 439 lesions with information on Fuhrman grade from 4 institutions were divided into training and test sets with an 8:2 split for model development and internal validation. Another 43 lesions from a separate institution were set aside for independent external validation. The performance of TPOT (Tree-Based Pipeline Optimization Tool), an automatic machine learning pipeline optimizer, was compared to hand-optimized machine learning pipeline. The best-performing hand-optimized pipeline was a Bayesian classifier with Fischer Score feature selection, achieving an external validation ROC AUC of 0.59 (95% CI 0.49–0.68), accuracy of 0.77 (95% CI 0.68–0.84), sensitivity of 0.38 (95% CI 0.29–0.48), and specificity of 0.86 (95% CI 0.78–0.92). The best-performing TPOT pipeline achieved an external validation ROC AUC of 0.60 (95% CI 0.50–0.69), accuracy of 0.81 (95% CI 0.72–0.88), sensitivity of 0.12 (95% CI 0.14–0.30), and specificity of 0.97 (95% CI 0.87–0.97). Automated machine learning pipelines can perform equivalent to or better than hand-optimized pipeline on an external validation test non-invasively predicting Fuhrman grade of renal cell carcinoma using conventional MRI.

## Introduction

Renal cell carcinoma (RCC) is the most prevalent renal malignancy in adults^1^. While the current standard for RCC management is partial or radical nephrectomy, the rising incidence of small RCC has led to the consideration of alternative treatment options for lower risk lesions, including percutaneous ablation and active surveillance^2^. Therefore, pre-treatment assessment of tumor aggressiveness is now of supreme importance for risk stratification and clinical decision making.

RCC outcome is closely linked to its pathological Fuhrman grade, which classifies RCC as low grade (Grade I–II) or high grade (Grade III–IV) according to the size, shape, staining, and presence or absence of nucleoli in the nuclei of cancer cells^3^. High-grade tumors are more invasive with metastasis potential and poor prognosis^4,5^. Biopsy is an invasive procedure with risk of complications and limited by the tumor heterogeneity^6^.

Recently, machine learning-based CT radiomics have been applied in prediction of Fuhrman grade with good results^7–9^. Radiomics, an emerging field in medical imaging, has grown exponentially for clinical decision support^10–12^. With a high volume of radiomic features extracted, feature choice in pipeline creation critically influences the results of final disease prediction or classification^12,13^. However, the selection of the most optimized pipeline requires extensive testing. The TPOT (Tree-Based Pipeline Optimization Tool) is an automated machine learning (autoML) that automatically chooses the most optimal machine learning pipeline and has been shown to outperform standard ML^14–16^.

The goal of the current study was to predict RCC grading using MR-based radiomics and compare performance of autoML with expert manual pipeline optimization on an external validation set.

## Methods and materials

### Patient cohort

Patients with histologically confirmed RCCs with available Fuhrman grading (I–IV) from two large academic centers in the United States (HUP and MAY), two hospitals in People’s Republic of China (SXH and PHH) and The Cancer Imaging Archive (TCIA) were retrospectively identified. The study was approved by the Institutional Review Boards of HUP, MAY, SXH, and PHH. With the agreement to use TCGA/TCIA data, the IRB approval of our study was waived for TCIA. The inclusion criteria were (1) pathologically confirmed RCC with reported histological Fuhrman grade (2) available preoperative MRI including T2-weighted (T2) and T1-contrast (T1C) enhanced sequences, (3) quality of the images was adequate for analysis, without motion or artifacts. The exclusion criteria consisted of (1) patients with WHO/ISUP grading (2) patients diagnosed through biopsy (3) patients with no reported Fuhrman grade (4) patients with incomplete or inappropriate image protocol. If dynamic enhancement was performed, the earliest phase on.

T1C sequence was chosen. Our final cohort consisted of 482 RCC lesions (374 lesions from HUP, 43 lesions from MAY, 39 lesions from TCIA, 11 lesions from SXH, and 15 lesions from PHH). Histopathological diagnosis in the form of Fuhrman grade was obtained for all 482 tumors after surgical excision. RCCs were grouped into low grade (grades I and II) and high grade (grades III and IV).

### Tumor segmentation

MR images of all patients were loaded into 3D Slicer software (v4.6), 3D regions of interest were manually drawn slice-by-slice on the T2 and T1C sequences by an abdominal radiologist (Y.Z.) with 5 years of experience reading abdominal MRI^17^.

### Image pre-processing

Preprocessing of the lesion images involved n4 bias correction and intensity normalization using ANTS and SimpleITK, respectively. The training set images were scaled to 200 by 200 pixel squares using bilinear interpolation, and augmented with horizontal/vertical flip, shear, and zoom transformations to add variability to the set.

### Training, validation, and testing

The 43 lesions from MAY were first separated out to use as our external testing set. The rest of the 439 lesions in our dataset were portioned into training and testing sets in a ratio of 8:2. Overall, the training set consisted of 351 lesions, the testing set consisted of 88 patients, and our external testing set consisted of 43 patients. The cohort can be seen in Table 1.

**Table 1:** Patient demographics, clinical features and tumor characteristics for overall cohort in training, validation, and test sets.

	Training set	Validation set	Test set	*P* value
	N = 351	N = 88	N = 43	
Age, median, range (years)	60.0 (27–92)	62.5 (30–81)	64.0 (38–85)	0.004*
Gender				0.092
Male	236 (67.2%)	61 (69.3%)	42 (82.4%)	
Female	115 (32.8%)	27 (30.7%)	9 (17.6%)	
Race				0.034*
White	241 (68.7%)	55 (62.5%)	45 (88.2%)	
Black	60 (17.1%)	21 (23.9%)	6 (11.8%)	
Asian	32 (9.1%)	9 (10.2%)	0 (18.1%)	
Unknown	18 (5.1%)	3 (3.4%)	0 (4.1%)	
Von Hipple– Lindau syndrome	6 (1.7%)	1 (1.1%)	0 (0%)	0.425
Subtype				< 0.001*
Clear cell	249 (71.0%)	66 (75.0%)	22 (43.1%)	
Papillary	78 (22.2%)	9 (10.2%)	22 (43.1%)	
Chromophobe	1 (0.3%)	3 (3.4%)	5 (9.8%)	
Clear cell papillary	15 (4.3%)	8 (9.1%)	0 (0%)	
Multilocular cystic	3 (0.9%)	1 (1.1%)	2 (3.9%)	
Unclassified	5 (1.4%)	1 (1.1%)	0 (0%)	
Laterality				0.820
Left	166 (47.3%)	40 (45.5%)	26 (51.0%)	
Right	185 (52.7%)	48 (54.5%)	25 (49.0%)	
Location				0.367
Upper	116(33.0%)	36 (40.9%)	13 (25.5%)	
Interpole	142 (40.5%)	33 (37.5%)	21 (41.2%)	
Lower	93 (26.5%)	19 (21.6%)	17 (33.3%)	
Tumor size, median, range (cm)	3.5 (0.9–18.7)	3.3 (1.0–17.2)	3.0 (0.2–15.5)	0.693
Renal vein invasion	36 (10.3%)	9 (10.2%)	0 (13.3%)	0.006*
Histological grade				0.351
Low grade	226 (64.4%)	59 (67.0%)	38 (74.5%)	
High grade	125 (35.6%)	29 (33.0%)	13 (25.5%)	
T stage				0.330
T1a	174 (49.6%)	48 (54.5%)	33 (64.7%)	
T1b	66 (18.8%)	15 (17.0%)	11 (21.6%)	
T2a	11 (3.1%)	2 (2.3%)	1 (2.0%)	
T2b	5 (1.4%)	2 (2.3%)	1 (2.0%)	
T3a	48 (13.7%)	11 (12.5%)	1 (2.0%)	
T3b	10 (2.8%)	1 (1.1%)	0 (0%)	
T3c	0 (0%)	0 (0%)	0 (0%)	
T4	0 (0%)	1 (1.1%)	0 (0%)	
Unavailable	37 (10.5%)	8 (9.1%)	4 (7.8%)	
Lymph node metastasis	5 (1.4%)	0 (0%)	0 (0%)	0.764
Distant metastasis	14 (4.0%)	5 (5.7%)	0 (0%)	0.094
Institution				< 0.001*
HUP	299 (85.2%)	75 (85. 2%)	0 (0%)	
SXY	8 (2.3%)	3 (3.4%)	0 (0%)	
PPH	12 (3.4%)	3 (3.4%)	0 (0%)	
TCIA	32 (9.1%)	7 (8.0%)	0 (0%)	
MAY	0 (0%)	0 (0%)	51 (100.0%)	

*Statistically significant.

### Radiomics analysis

Radiomics features were extracted from each patient’s MRI for both T1C and T2WI sequences. For each image space, 79 non-texture (morphology and intensity-based) and 94 texture features were extracted according to the guidelines defined by the Image Biomarker Standardization Initiative (IBSI)^18^. Each of the 94 texture features were computed 32 times using all possible combinations of the following extraction parameters, a process known as “texture optimization” (REF): (1) isotropic voxels of size 1 mm, 2 mm, 3 mm, and 4 mm, (2) fixed bin number (FBN) discretization algorithm, with and without equalization, and (3) the number of gray levels of 8, 16, 32, and 64 for FBN. A total of (79 + 32 × 94), or 3087, radiomics features were thus computed in this study. All the features were normalized using unity-based normalization and features from T1C and T2WI were combined into one dataset. In order to reduce dimensionality of the datasets, radiomics features were selected for training using thirteen different feature selection methods. Ten machine learning classifiers were trained and tested on features from the same splits of patients used in the deep learning methods. The detailed feature selection methods and classifiers used are shown in Supplementary Table 1. Each classifier was trained on the training set thirteen times using thirteen different feature selection methods and validated through tenfold cross-validation. Classifiers were trained on 10, 30, 50, and 100 selected features and performances were compared on the testing set. In addition to performance, the stability of both classifiers and feature selection methods was recorded. Relative standard deviation (RSD%) was calculated for classifier stability. Each classifier was trained and validated on different sub-samples of the data 100 times, and RSD % was calculated by the standard deviation of AUC divided by the mean of AUC for these 100 trials. A stability measure proposed by Nogueira et al. was used for feature selection stability^19^. This function quantified stability of feature selection as the similarity between selected feature sets obtained by the same method over multiple trials. The same feature selection method was run on varying sets of training data 100 times, selecting 50 features at a time. With this data, the stability function outputted a number between 0 and 1, where 1 is most stable, or least variance between selected features and 0 is least stable, or most variance between selected features. The performance of the top-performing classifier was then compared to the performance of an automated optimized machine learning pipeline computed by TPOT, a Tree-Based Pipeline Optimization Tool that chooses the most optimal machine learning pipeline for an inputted dataset through genetic programming. To reduce stochasticity, 10 iterations of the TPOT software were run on the training and testing sets. The best-performing hand-optimized model and the best-performing TPOT pipeline were then tested on the final external testing set.

### Statistical analysis

For the radiomics analysis methods, the following performance metrics were calculated: accuracy, sensitivity, specificity, and area under Receiver Operating Characteristic curve (ROC AUC). In addition, the median, mean, and standard deviation ROC AUC was calculated for each classifier’s performance on the testing set. The ROC curve and Precision-Recall curve were plotted to measure the performance of the binary classifiers. Average accuracy, sensitivity, and specificity with 95% confidence interval were calculated using the adjusted Wald method. The p-values quantifying the differences in performance between the TPOT and hand-optimized pipelines were calculated using the binomial test for specificity, sensitivity, and accuracy and the Wilcoxon test for ROC-AUC^20^.

### Code availability

The implementation of the radiomics feature extraction was based on “radiomics-develop” package of McGill University^21,22^. This code is available for public use on Github at https://github.com/mvallieres/radiomics-develop. The auto-ML script utilized the TPOT package from the Epistasis Lab and can be found at https://github.com/EpistasisLab/tpot. The implementation of the machine learning models was based on the sklearn package of Python. To allow others to develop similar models, the code is publicly available at https://github.com/subhanik1999/Radiomics-ML.

## Results

### Patient and tumor characteristics

Supplementary Table 2 shows the clinicopathologic characteristics of our cohort. High grade RCCs were significantly larger than low grade RCCs (mean size, 4.9 cm vs. 2.7 cm, *p* < 0.001). Renal vein invasion was found in 34 high-grade RCC lesions, whereas only 11 low grade RCC lesions presented with this feature (*p* < 0.001). There was significant difference in T stage between the two groups (*p* < 0.001). Presence of lymph node involvement and distance metastasis were more common in high-grade RCC than low-grade RCC (*p* = 0.004, and *p* = 0.001, respectively).

**Table 2:** Comparison results of 10 TPOT models.

Model index	AUC	Accuracy	Sensitivity	Specificity	Precision	Hamming loss	Kappa
1	0.52	0.58	0.11	0.92	0.43	0.42	0.03
2	0.65	0.73	0.46	0.85	0.43	0.27	0.33
3	0.65	0.75	0.38	0.92	0.44	0.25	0.34
4	0.65	0.75	0.38	0.92	0.44	0.25	0.34
5	0.63	0.73	0.38	0.88	0.41	0.28	0.29
6^a^	0.67	0.76	0.42	0.92	0.47	0.24	0.38
7	0.61	0.71	0.35	0.86	0.38	0.29	0.23
8	0.55	0.71	0.15	0.95	0.35	0.29	0.13
9	0.65	0.75	0.38	0.92	0.44	0.25	0.34
10	0.61	0.73	0.31	0.92	0.40	0.27	0.26

^a^Model 6 was selected as the final TPOT model for further external validation.

### Internal testing results

**Figure 1:**
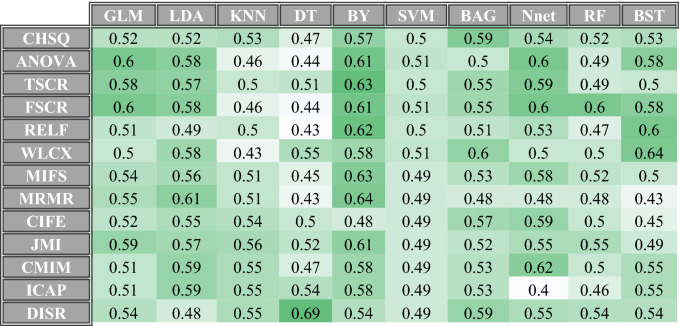
Heatmap of ROC-AUCs on internal validation set of classifier and feature selection combinations for 50 selected features.

The radiomics analysis showed that the Bayesian classifier (BY) had the highest median and mean validation ROC AUC scores in predicting the grade of renal tumors. Specifically, BY achieved a median ROC AUC of 0.61 (95% CI 0.51–0.70) and a mean ROC AUC of 0.60 (95% CI 0.50–0.69). The median and mean ROC AUCs for all the classifiers are shown in Supplementary Table 4. A heatmap displaying the validation ROC AUCs of the classifier and feature selection methods on 50 selected features is shown in Fig. 1. The Fischer score (FSCR) feature selection method corresponded to the highest median and mean validation ROC AUC among classifiers. Specifically, FSCR corresponded to a median validation ROC AUC of 0.58 (95% CI 0.48–0.67) and mean ROC AUC of 0.56 (95% CI 0.46–0.65). The median and mean ROC AUCs for all the feature selection methods are shown in Supplementary Table 5. Stability measures of all the classifiers and feature selection methods are shown in Supplementary Tables 6 and 7. The TPOT pipeline specifics are shown in Supplementary Table 8. Out of the 10 TPOT pipelines, Pipeline 6 had the highest median and mean validation ROC AUC. The RandomForestClassifier exported by TPOT in Pipeline 6 achieved a validation ROC AUC of 0.67 (95% CI 0.57–0.75) as shown in Table 2. The validation performances of the other 9 TPOT pipelines are also shown in Table 2. The performance of BY was compared to the performance of the Pipeline 6 exported by TPOT. In comparing the internal testing results, the Bayesian classifier’s best performance produced a slightly higher validation ROC AUC than that of the best TPOT-exported pipeline (0.68 vs. 0.67). Heatmap of ROC-AUCs on internal testing set of classifier and feature selection combinations for 10, 30, 100 selected features were shown in Supplementary Figs. 1–3.

### External testing results

**Figure 2:**
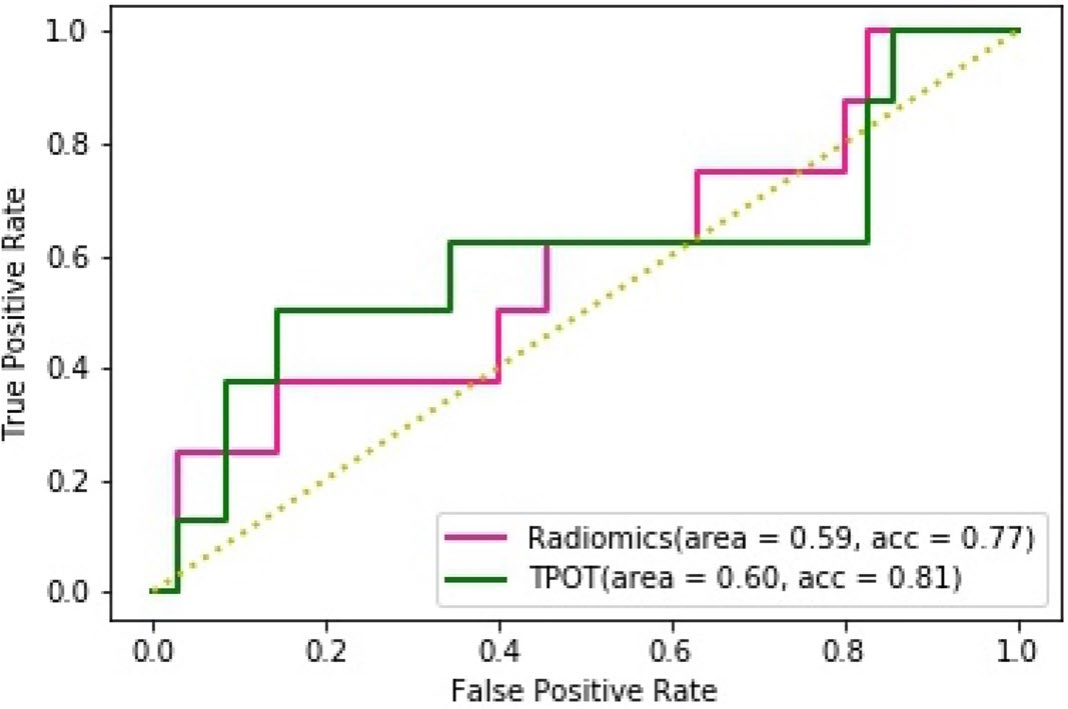
ROC curve plotted for the hand-optimized radiomics pipeline and the TPOT pipeline on the external test set.

As the top-performing classifier and feature selection model, BY and FSCR were then tested on the final external testing set. This hand-optimized pipeline achieved a test ROC AUC of 0.59 (95% CI 0.49–0.68), accuracy of 0.77 (95% CI 0.68–0.84), sensitivity of 0.38 (95% CI 0.29–0.48), and a specificity of 0.86 (95% CI 0.78–0.92). The top-performing TPOT exported pipeline was also tested on the external test set for comparison. This pipeline achieved a test ROC AUC of 0.60 (95% CI 0.50–0.69), accuracy of 0.81 (95% CI 0.72–0.88), sensitivity of 0.12 (95% CI 0.14–0.30), and specificity of 0.97 (95% CI 0.87–0.97). In comparing the performance of the TPOT-exported pipeline to the BY/FSCR pipeline, the TPOT pipeline achieved a higher test ROC AUC (0.60 vs. 0.59, *p* = 0.94), a higher test accuracy (0.81 vs. 0.77, *p* = 0.71), a lower sensitivity (0.13 vs. 0.38, *p* = 0.07), and a higher specificity (0.97 vs. 0.86, *p* = 0.004). The ROC curves for both the manual expert-optimized and TPOT pipelines are shown in Fig. 2.

## Discussion

Prior to the development of our ML-based MRI radiomics study, manual MRI characteristics, such as apparent diffusion coefficient (ADC) measurements, have been used to differentiate low and high grade RCC. In such cases, ADC measurements are taken using different region-of-interest (ROI) placement techniques to distinguish between low and high grade. A study using ADC differentiation by Aslan et al. demonstrates an accuracy value of 0.75 whereas our study displays a top accuracy of 0.81^23^. ML-based radiomics methods have potential to enhance differentiation based on grade compared to manual ADC computation. In this study, we specifically compared automatic and manually optimized machine learning pipeline using MR-based radiomics in discriminating between low and high grade RCCs. The TPOT-exported pipeline and the top manually optimized pipeline achieved similar accuracy. When a machine learning model is implemented as a tool for RCC risk stratification, high specificity is the most important performance measure. With higher specificity, low risk patients can potentially be offered less invasive alternative treatment to preserve renal function and minimize long-term complications. In our study, TPOT generated a pipeline which had higher specificity than the top manually optimized pipeline on the external test set.

Previous studies have investigated the value of CT-based radiomics in distinguishing low from high Fuhrman grade RCC^7–9^. Texture features can quantify tumor heterogeneity and were found to be correlated with Fuhrman grade^24,25^. Shu et al. selected CT radiomics features from corticomedullary (CMP) and nephrographic (NP) phase using least absolute shrinkage and selection operator (LASSO) and constructed logistic regression model to discriminate between high and low grades. The model combining the features from both CMP and NP achieved the highest accuracy of 0.78 and ROC of 0.82^8^. Ding et al. used similar method to build a CT-radiomics based predictive model identifying 145 high-grade RCC from 61 low-grade RCCs with an AUC of 0.88 in training cohort and 0.77 in testing cohort^7^. Bektas et al. combined 5 machine learning classifiers with wrapper-based feature selection on texture features to differentiate 31 low-grade from 23 high-grade RCCs. The best model created using support vector machine achieved an accuracy of 0.85 and ROC of 0.86^9^. The referenced studies proved CT radiomics was useful and promising for non-invasive prediction of Fuhrman grade, but due to only having a cohort from a single institution, these predictive models were not validated externally in an independent cohort, which makes generalization questionable. However, since we do not have the datasets or code of these referenced studies, we cannot make a direct comparison on performance. Overall, our study rigorously evaluated a variety of machine learning approaches and included external validation to assess for expected performance on deployment. Additionally, there are a few quantitative differences in the methodologies used in our study and those used in the studies above. Compared to our external test set of 43 patients, these studies predicted Fuhrman grade on a greater number of patients i.e. 92, 260, and 54. Additionally, these studies utilized more targeted feature selection methodology, computing interclass correlation coefficients (ICC) between feature types, resulting in a significantly fewer number of selected features i.e. 13, 35, and 4. The hand-optimized pipeline in our study selected 50 features through a holistic statistical approach on all feature types, thus streamlining the process, reducing feature bias, but potentially affecting the performance.

Compared with previous radiomics studies, our study has several differences. First, we chose MRI instead of CT. MRI provides multi-parametric sequence, which theoretically provide more information than simple attenuation differences measured in Hounsfield units on CT. Second, we have investigated and compared a large group of feature selection methods and classifiers for radiomics-based Fuhrman grade prediction, and the model with highest performance was then compared with an automated optimized machine learning pipeline computed by TPOT. Third, our cohort come from five institutions, one of which was separated as an independent test set to implement external validation strategy, which none of the previous studies have attempted.

In this study, we investigated 13 different filter-based feature selection methods and 10 machine-learning classification methods belonging to 10 different classifier families. We only used filter-based approaches because they are computationally more efficient and less prone to overfitting than the wrapper and embedded methods^26,27^. Furthermore, filter methods are classifier independent, which allow separation of the feature selection and modeling and could increase the generalizability of each component and hence the overall analysis^12^. Our results show that the Bayesian classifier yields the highest predictive performance among the 10 classifiers. Bayesian classifier is fast and simple to train and good at dealing with small data, but have difficulties with complex datasets and shows inferior performance on large datasets^28,29^. The best TPOT-exported pipeline was created using random forest. Random forests have become particularly popular, due to several advantages that include fast training times, the ability to use high dimensional data (where number of features are significantly larger than the number of patients) and high generalizability, but it has been observed to have a problem with overfitting^12,30^. The best TPOT-exported pipeline performed similarly to the top manually optimized pipeline on the internal test set. On the external test set, both pipelines experienced a slight dip in performance, but TPOT slightly outperformed the manually optimized pipeline. The TPOT performance on the external test set is a strength of our study, suggesting that the performance of autoML may be more generalizable.

Limitations of this study include the retrospective selection of only patients with available Fuhrman grade, which may have resulted in selection bias. Second, Fuhrman grade was determined as recorded in the pathology report of the original pathologist. Review by additional pathologists was not feasible due to missing slides and limited resources. Third, segmentation was performed by a single radiologist with 5 years of experience. Automatic renal tumor segmentation will be incorporated in future work. Fourth, the performance was still suboptimal for real-time clinical use. However, the main goal of our paper was to compare the performance of autoML with that manual expert optimized pipeline on external testing.

In this study, TPOT was shown to differentiate low from high histological grade RCC with performance metrics that are slightly better than expert manual pipeline optimization on an external validation set. These results suggest that autoML-based radiomics based on MRI, without the requirement of a machine learning expert, may be a valid strategy to predict RCC characteristics.

## Supplementary information

Supplementary information.
