# Supplementary material for: Differentiation of low and high grade renal cell carcinoma on routine MRI with an externally validated automatic machine learning algorithm
Source: Sci Rep. 2020 Nov 11;10:19503. doi: 10.1038/s41598-020-76132-z (PMC7658976; doi:10.1038/s41598-020-76132-z)
Supplement: Supplementary file 1 — Supplementary information. [file 41598_2020_76132_MOESM1_ESM.docx]

**Title:**

Differentiation of low and high grade renal cell carcinoma on routine MRI with an externally validated automatic machine learning algorithm

**Authors:**

Subhanik Purkayastha^1*^, Yijun Zhao^2*^, Jing Wu^2^, Rong Hu^8^, Aidan McGirr^4^, Sukhdeep Singh^4^, Ken Chang^5^, Raymond Y. Huang^6^, Paul J. Zhang^7^, Alvin Silva^4^, Michael C. Soulen^3^, S. William Stavropoulos^3^, Zishu Zhang^2^, Harrison X. Bai^1^

1. Department of Diagnostic Imaging, Rhode Island Hospital and Alpert Medical School of Brown University, Providence, RI, USA

2. Department of Radiology, The Second Xiangya Hospital, Central South University, Changsha, China

3. Division of Interventional Radiology, Department of Radiology, Hospital of the University of Pennsylvania, PA, USA

4. Department of Radiology, Mayo Clinic, Phoenix, Arizona, USA

5. Athinoula A. Martinos Center for Biomedical Imaging, Department of Radiology, Massachusetts General Hospital, Boston, MA, USA

6. Department of Radiology, Brigham and Women's Hospital, Boston, MA, USA

7. Department of Pathology, Hospital of the University of Pennsylvania, Philadelphia, PA, USA

8. School of Computer Science and Engineering, Central South University, Changsha, Hunan, China

**Supplementary Table 1. Table of abbreviations and names of classification and feature selection methods**

| Classification Method Acronym | Classification Method Name | Feature Selection Method Acronym | Feature Selection Method Name |
| --- | --- | --- | --- |
| Nnet | Neural Network | CHSQ | Chi-square Score |
| DT | Decision Tree | ANOVA | Analysis of Variance |
| BST | Boosting | TSCR | T-test Score |
| BY | Bayesian | FSCR | Fischer Score |
| BAG | Bagging | RELF | Relief |
| RF | Random Forest | WLCX | Wilcoxon |
| SVM | Support Vector Machines | MIFS | Mutual Information Feature Selection |
| LDA | Linear Discriminant Analysis | MRMR | Minimum Redundancy Maximum Relevance |
| KNN | k-Nearest Neighbors | CIFE | Conditional Infomax Feature Selection |
| GLM | Generalized Linear Models | JMI | Joint Mutual Information |
| - | - | CMIM | Conditional Mutual Information Maximization |
| - | - | ICAP | Interaction Capping |
| - | - | DISR | Double Input Symmetric Relevance |

**Supplementary Table 2. Patient demographics, clinical features and tumor characteristics between low and high histological grade groups**

|  | Low grade  (grade I and II)  N = 323 | High grade  (grade III and IV)  N = 167 | P value |
| --- | --- | --- | --- |
| Age, median, range (years) | 61.0 (27-85) | 62.0 (28-92) | 0.401 |
| Gender |  |  | 0.031* |
| Male | 213 (65.9%) | 126 (75.4%) |  |
| Female  Race  White  Black  Asian  Unknown  Von Hipple-Lindau syndrome | 110 (34.1%)  212 (65.6%)  65 (20.1%)  33 (10.3%)  13 (4.0%)  0 (2.2%) | 41 (24.6%)  129 (77.2%)  22 (13.2%)  8 (4.8%)  8 (4.8%)  0 (0%) | 0.038*  0.101 |
| Subtype |  |  | 0.011* |
| Clear cell | 227 (70.3%) | 110 (65.9%) |  |
| Papillary | 62 (19.2%) | 47 (28.1%) |  |
| Chromophobe | 8 (2.5%) | 1 (0.6%) |  |
| Clear cell papillary | 18 (5.6%) | 5 (3.0%) |  |
| Multilocular cystic | 6 (1.9%) | 0 (0%) |  |
| Unclassified | 2 (0.6%) | 4 (2.4%) |  |
| Laterality |  |  | 0.859 |
| Left | 152 (47.1%) | 80 (47.9%) |  |
| Right | 171 (52.9%) | 87 (52.1%) |  |
| Location |  |  | 0.414 |
| Upper | 110 (34.1%) | 55 (32.9%) |  |
| Interpole | 123 (38.1%) | 73 (43.7%) |  |
| Lower  Tumor size, median, range (cm)  Renal vein invasion | 90 (27.8%)  2.7 (0.2-18.7)  11 (3.4%) | 39 (23.4%)  4.9 (1.1-18.1)  34 (20.4%) | <0.001*  <0.001* |
| T stage  T1a  T1b  T2a  T2b | 203 (62.8%)  55 (17.0%)  5 (1.5%)  6 (1.9%) | 52 (31.1%)  37 (22.2%)  9 (5.4%)  2 (1.2%) | <0.001* |
| T3a  T3b  T3c  T4  Unavailable  Lymph node metastasis  Distant metastasis | 17 (5.3%)  3 (0.9%)  0 (0%)  0 (0%)  34 (10.5%)  0 (0%)  6 (1.9%) | 43 (25.7%)  8 (4.8%)  0 (0%)  1 (0.6%)  15 (9.0%)  5 (3.0%)  13 (7.8%) | 0.004*  0.001* |

* Statistically significant

**Supplementary Table 3. MR scanning parameters in HUP, MAY, SXY, PHH and TCIA cohort for T1C and T2 sequences.**

|  | HU | MA | SX | PH | TCIA |
| --- | --- | --- | --- | --- | --- |
| Magnetic fields, median, range (T) | 1.7 (1.0-3.0) | 2.2 (1.5-3.0) | 3.0 (3.0-3.0) | 3.0 (1.5-3.0) | 1.5 (1.0-3.0) |
| T1C sequence |  |  |  |  |  |
| Breathing techniques  Fat-suppression, n (%)  TR, mean, range (ms) | Breath-hold  343 (100.0%)  34.7 (2.6-600.0) | Breath-hold  48(100.0%)  8.6(3.0-240.0) | Breath-hold  10(100.0%)  3.9 (3.0-3.9) | Breath-hold  15(100.0%)  3.4 (3.4-3.9) | Breath-hold  14(100.0%)  4.7 (3.0-260.0) |
| TE, median, range (ms)  SL, median, range (mm)  Voxel size, median, range (mm^3^)  T2 sequence  Breathing techniques  Fat-suppression, n (%)  TR, median, range (ms)  TE, median, range (ms)  SL, median, range (mm)  Voxel size, median, range (mm^3^) | 2.1 (0.8-21.0)  4.3 (1.8-8.0)  1.0×1.0×4.3（0.5×0.5×1.8-2.0×2.0×8.0）  Breath-hold  123 (35.9%)  2065.3  (3.0-34105.4)  108.0 (1.1-402.2)  6.5 (4.0-8.0)  1.2×1.2×6.5 (0.6×0.6×4.0-2.0×2.0×8.0) | 1.7 (1.2-4.2)  3.4 (1.3-7.0)  1.1×1.1×3.4（0.5×0.5×1.3-1.5×1.5×7.0）  Breath-hold  35(72.9%)  2560.9  (1200.0-15000.0)  91.3 (63.0-112.0)  5.8 (4.0-8.0)  1.1×1.1×5.8 (0.7×0.7×4.0-1.8×1.8×8.0) | 1.8 (1.4-1.9)  3.0 (1.5-4.0)  1.3×1.3×3.0（0.6×0.6×1.5-1.5×1.5×4.0）  Breath-hold  10(100.0%)  1600.0  (1113.1-1600.0)  91.0 (70.0-93.0)  5.0 (3.3-6.0)  1.2×1.2×5.0 (0.7×0.7×3.3-1.2×1.2×6.0) | 1.2 (1.2-1.8)  2.9 (2.5-6.5)  1.6×1.6×2.9 (1.5×1.5×2.5-2.0×2.0×6.5)  Breath-hold  14(93.3%)  3091.1  (1032.5-5059.5)  83.0 (80.0-96.0)  5.0 (4.0-6.5)  0.8×0.8×5.0 (0.6×0.6×4.0-1.4×1.4×6.5) | 2.0 (0.8-13.0)  5.0 (1.5-10.0)  1.1×1.1×5.0 (0.6×0.6×1.5-1.6×1.6×5.0)  Breath-hold  8(57.1%)  2400.0  (3.8-9230.8)  97.6 (1.7-181.4)  7.0 (4.0-8.0)  1.3×1.3×7.0 (0.6×0.6×4.0-1.6×1.6×8.0) |

*T1C, T1-contrast; TR, Repetition time; TE, Echo time; SL, Slice thickness

**Supplementary Table 4. Median and mean AUC and standard deviation of AUC for each classifier on internal validation set**

| Classifier | Median AUC | Mean AUC | St. Deviation |
| --- | --- | --- | --- |
| GLM | 0.55 | 0.55 | 0.04 |
| NNet | 0.58 | 0.56 | 0.06 |
| KNN | 0.55 | 0.55 | 0.04 |
| BST | 0.56 | 0.55 | 0.06 |
| BAG | 0.53 | 0.54 | 0.04 |
| RF | 0.54 | 0.53 | 0.04 |
| LDA | 0.57 | 0.56 | 0.05 |
| BY | 0.61 | 0.60 | 0.04 |
| DT | 0.51 | 0.52 | 0.07 |
| SVM | 0.49 | 0.50 | 0.01 |

**Supplementary Table 5. Median and mean AUC and standard deviation of AUC for each feature selection method on internal validation set**

| Feature Selection | Median AUC | Mean AUC | St. Deviation |
| --- | --- | --- | --- |
| CHSQ | 0.52 | 0.53 | 0.03 |
| ANOVA | 0.54 | 0.54 | 0.06 |
| TSCR | 0.53 | 0.54 | 0.05 |
| FSCR | 0.58 | 0.56 | 0.06 |
| RELF | 0.51 | 0.52 | 0.06 |
| WLCX | 0.53 | 0.54 | 0.06 |
| MIFS | 0.53 | 0.53 | 0.05 |
| MRMR | 0.49 | 0.51 | 0.07 |
| CIFE | 0.51 | 0.52 | 0.04 |
| JMI | 0.55 | 0.55 | 0.04 |
| CMIM | 0.54 | 0.54 | 0.05 |
| ICAP | 0.54 | 0.52 | 0.06 |
| DISR | 0.54 | 0.55 | 0.06 |

**Supplementary Table 6. Stability of classifiers using relative standard deviation percent (RSD %) of AUC**

| Classifier | RSD% |
| --- | --- |
| GLM | 4.98 |
| NNet | 6.57 |
| KNN | 4.16 |
| BST | 5.46 |
| BAG | 4.33 |
| RF | 4.30 |
| LDA | 4.65 |
| BY | 10.15 |
| DT | 5.70 |
| SVM | 0.63 |

**Supplementary Table 7. Stability of feature selection methods using stability function by *Nogueira et al.***

| Feature Selection | Stability |
| --- | --- |
| CHSQ | 0.861 |
| ANOVA | 0.799 |
| TSCR | 0.754 |
| FSCR | 0.796 |
| RELF | 0.489 |
| WLCX | 0.767 |
| MIFS | 0.998 |
| MRMR | 0.983 |
| CIFE | 0.943 |
| JMI | 0.161 |
| CMIM | 0.658 |
| ICAP | 0.674 |
| DISR | 0.683 |

**Supplementary Table 8. TPOT exported pipelines from 10 iterations of the software on the training and validation sets**

| Pipeline Number | Parameters |
| --- | --- |
| 1 | RandomForestClassifier(input_matrix, bootstrap=False, criterion=gini, max_features=0.1, min_samples_leaf=16, min_samples_split=5, n_estimators=100) |
| 2 | RandomForestClassifier(CombineDFs(KNeighborsClassifier(input_matrix, n_neighbors=11, p=1, weights=uniform), input_matrix), bootstrap=True, criterion=gini, max_features=0.8, min_samples_leaf=17, min_samples_split=16, n_estimators=100) |
| 3 | ExtraTreesClassifier(ZeroCount(MinMaxScaler(SelectPercentile(input_matrix, percentile=19))), bootstrap=True, criterion=gini, max_features=0.3, min_samples_leaf=12, min_samples_split=12, n_estimators=100) |
| 4 | ExtraTreesClassifier(OneHotEncoder(input_matrix, minimum_fraction=0.15, sparse=False, threshold=10), bootstrap=True, criterion=entropy, max_features=0.4, min_samples_leaf=6, min_samples_split=12, n_estimators=100) |
| 5 | ExtraTreesClassifier(CombineDFs(input_matrix, input_matrix), bootstrap=True, criterion=entropy, max_features=0.7000000000000001, min_samples_leaf=19, min_samples_split=2, n_estimators=100) |
| 6 | RandomForestClassifier(MaxAbsScaler(input_matrix), bootstrap=True, criterion=gini, max_features=0.9000000000000001, min_samples_leaf=17, min_samples_split=6, n_estimators=100) |
| 7 | ExtraTreesClassifier(input_matrix, bootstrap=True, criterion=entropy, max_features=0.2, min_samples_leaf=5, min_samples_split=3, n_estimators=100) |
| 8 | GradientBoostingClassifier(input_matrix, learning_rate=0.01, max_depth=7, max_features=0.5, min_samples_leaf=16, min_samples_split=20, n_estimators=100, subsample=0.35000000000000003) |
| 9 | ExtraTreesClassifier(CombineDFs(input_matrix, input_matrix), bootstrap=True, criterion=entropy, max_features=0.8, min_samples_leaf=11, min_samples_split=2, n_estimators=100) |
| 10 | ExtraTreesClassifier(CombineDFs(input_matrix, input_matrix), bootstrap=True, criterion=entropy, max_features=0.3, min_samples_leaf=11, min_samples_split=18, n_estimators=100) |
|  |  |

|  | **GLM** | **LDA** | **KNN** | **DT** | **BY** | **SVM** | **BAG** | **Nnet** | **RF** | **BST** |
| --- | --- | --- | --- | --- | --- | --- | --- | --- | --- | --- |
| **CHSQ** | 0.52 | 0.54 | 0.5 | 0.51 | 0.55 | 0.5 | 0.47 | 0.52 | 0.51 | 0.54 |
| **ANOVA** | 0.6 | 0.59 | 0.58 | 0.51 | 0.62 | 0.51 | 0.52 | 0.59 | 0.59 | 0.6 |
| **TSCR** | 0.6 | 0.6 | 0.57 | 0.46 | 0.67 | 0.5 | 0.55 | 0.6 | 0.56 | 0.56 |
| **FSCR** | 0.6 | 0.59 | 0.58 | 0.64 | 0.62 | 0.51 | 0.49 | 0.58 | 0.54 | 0.6 |
| **RELF** | 0.51 | 0.54 | 0.59 | 0.55 | 0.68 | 0.5 | 0.59 | 0.52 | 0.54 | 0.57 |
| **WLCX** | 0.49 | 0.57 | 0.53 | 0.62 | 0.58 | 0.45 | 0.51 | 0.53 | 0.56 | 0.54 |
| **MIFS** | 0.5 | 0.5 | 0.54 | 0.55 | 0.58 | 0.49 | 0.51 | 0.46 | 0.55 | 0.5 |
| **MRMR** | 0.5 | 0.5 | 0.53 | 0.53 | 0.58 | 0.49 | 0.54 | 0.66 | 0.48 | 0.48 |
| **CIFE** | 0.51 | 0.5 | 0.55 | 0.53 | 0.5 | 0.49 | 0.49 | 0.5 | 0.51 | 0.55 |
| **JMI** | 0.53 | 0.54 | 0.55 | 0.5 | 0.53 | 0.49 | 0.53 | 0.52 | 0.55 | 0.61 |
| **CMIM** | 0.51 | 0.52 | 0.55 | 0.41 | 0.53 | 0.49 | 0.5 | 0.52 | 0.5 | 0.56 |
| **ICAP** | 0.51 | 0.52 | 0.55 | 0.47 | 0.53 | 0.49 | 0.5 | 0.5 | 0.5 | 0.56 |
| **DISR** | 0.51 | 0.52 | 0.55 | 0.44 | 0.53 | 0.49 | 0.49 | 0.5 | 0.52 | 0.54 |

**Supplementary Figure 1. Heatmap of ROC-AUCs on internal validation set of classifier and feature selection combinations for 10 selected features**

**Supplementary Figure 2. Heatmap of ROC-AUCs on internal validation set of classifier and feature selection combinations for 30 selected features**

|  | **GLM** | **LDA** | **KNN** | **DT** | **BY** | **SVM** | **BAG** | **Nnet** | **RF** | **BST** |
| --- | --- | --- | --- | --- | --- | --- | --- | --- | --- | --- |
| **CHSQ** | 0.52 | 0.49 | 0.54 | 0.47 | 0.55 | 0.5 | 0.57 | 0.57 | 0.56 | 0.51 |
| **ANOVA** | 0.57 | 0.51 | 0.53 | 0.5 | 0.59 | 0.51 | 0.49 | 0.62 | 0.57 | 0.52 |
| **TSCR** | 0.56 | 0.47 | 0.52 | 0.43 | 0.63 | 0.5 | 0.45 | 0.59 | 0.5 | 0.49 |
| **FSCR** | 0.57 | 0.51 | 0.53 | 0.48 | 0.6 | 0.51 | 0.54 | 0.6 | 0.54 | 0.52 |
| **RELF** | 0.54 | 0.55 | 0.55 | 0.52 | 0.64 | 0.5 | 0.51 | 0.54 | 0.55 | 0.57 |
| **WLCX** | 0.55 | 0.52 | 0.58 | 0.48 | 0.59 | 0.53 | 0.55 | 0.5 | 0.49 | 0.58 |
| **MIFS** | 0.52 | 0.6 | 0.51 | 0.55 | 0.67 | 0.49 | 0.5 | 0.5 | 0.5 | 0.48 |
| **MRMR** | 0.54 | 0.53 | 0.51 | 0.53 | 0.65 | 0.49 | 0.56 | 0.5 | 0.49 | 0.54 |
| **CIFE** | 0.59 | 0.56 | 0.54 | 0.54 | 0.5 | 0.49 | 0.53 | 0.5 | 0.57 | 0.5 |
| **JMI** | 0.57 | 0.48 | 0.59 | 0.53 | 0.65 | 0.49 | 0.53 | 0.59 | 0.49 | 0.47 |
| **CMIM** | 0.52 | 0.52 | 0.5 | 0.43 | 0.6 | 0.49 | 0.5 | 0.54 | 0.49 | 0.5 |
| **ICAP** | 0.52 | 0.52 | 0.5 | 0.52 | 0.6 | 0.49 | 0.52 | 0.62 | 0.6 | 0.5 |
| **DISR** | 0.58 | 0.53 | 0.55 | 0.47 | 0.53 | 0.49 | 0.49 | 0.57 | 0.55 | 0.47 |

**Supplementary Figure 3. Heatmap of ROC-AUCs on internal validation set of classifier and feature selection combinations for 100 selected features**

|  | **GLM** | **LDA** | **KNN** | **DT** | **BY** | **SVM** | **BAG** | **Nnet** | **RF** | **BST** |
| --- | --- | --- | --- | --- | --- | --- | --- | --- | --- | --- |
| **CHSQ** | 0.52 | 0.56 | 0.58 | 0.54 | 0.57 | 0.5 | 0.53 | 0.54 | 0.48 | 0.53 |
| **ANOVA** | 0.6 | 0.59 | 0.54 | 0.43 | 0.61 | 0.51 | 0.5 | 0.59 | 0.48 | 0.58 |
| **TSCR** | 0.58 | 0.56 | 0.47 | 0.42 | 0.65 | 0.5 | 0.5 | 0.58 | 0.54 | 0.58 |
| **FSCR** | 0.6 | 0.59 | 0.54 | 0.42 | 0.61 | 0.51 | 0.55 | 0.59 | 0.52 | 0.58 |
| **RELF** | 0.53 | 0.51 | 0.55 | 0.53 | 0.67 | 0.5 | 0.56 | 0.58 | 0.57 | 0.55 |
| **WLCX** | 0.5 | 0.6 | 0.45 | 0.63 | 0.57 | 0.48 | 0.54 | 0.52 | 0.53 | 0.55 |
| **MIFS** | 0.52 | 0.54 | 0.51 | 0.49 | 0.65 | 0.49 | 0.5 | 0.48 | 0.52 | 0.46 |
| **MRMR** | 0.5 | 0.5 | 0.51 | 0.43 | 0.58 | 0.49 | 0.5 | 0.48 | 0.5 | 0.47 |
| **CIFE** | 0.53 | 0.51 | 0.54 | 0.55 | 0.51 | 0.49 | 0.58 | 0.4 | 0.57 | 0.48 |
| **JMI** | 0.57 | 0.58 | 0.55 | 0.56 | 0.5 | 0.49 | 0.58 | 0.58 | 0.52 | 0.56 |
| **CMIM** | 0.53 | 0.51 | 0.55 | 0.45 | 0.6 | 0.49 | 0.44 | 0.6 | 0.53 | 0.52 |
| **ICAP** | 0.53 | 0.51 | 0.55 | 0.49 | 0.6 | 0.49 | 0.51 | 0.58 | 0.54 | 0.52 |
| **DISR** | 0.56 | 0.54 | 0.55 | 0.49 | 0.51 | 0.49 | 0.47 | 0.57 | 0.53 | 0.5 |
